# Physical activity patterns in older men and women in Germany: a cross-sectional study

**DOI:** 10.1186/1471-2458-11-559

**Published:** 2011-07-13

**Authors:** Anna Moschny, Petra Platen, Renate Klaaßen-Mielke, Ulrike Trampisch, Timo Hinrichs

**Affiliations:** 1 Department of Sports Medicine and Sports Nutrition, Ruhr-University Bochum, Germany; 2 Department of Medical Informatics, Biometry and Epidemiology, Ruhr-University Bochum, Germany

**Keywords:** Aged, gender, physical activity, housework, gardening, correlates, public health

## Abstract

**Background:**

Data on physical activity in older adults in Germany is scarce. The aim of this study was to analyze physical activity patterns and to explore factors associated with physical activity in different domains, i.e. sporting activities (SA) and domestic activities (DA), in older men and women.

**Methods:**

As part of the 7-year follow-up telephone interviews of the getABI cohort (community-dwelling older adults in Germany), the PRISCUS-PAQ was used to survey participants about their everyday physical activity patterns. Time per week (hh:mm) spent in SA and DA (heavy housework, gardening) was analyzed for men and women. Multivariate logistic regression analyses were performed in order to assess the odds of participating in SA and DA for at least 2.5 hours/week in association with sociodemographic factors, a broad range of physical health-related factors and interview date (season of the year).

**Results:**

A total of 1,610 primary health care patients (51.6% women) with a median age of 77 (range 72-93) years were included in the analyses. Men engaged in SA more often than women (01:45 vs. 01:10), whereas women did more DA per week than men (04:00 vs. 03:00).

Being interviewed in spring or summer was associated with increased performance of DA in both sexes. Participation in these activities was reduced in more highly educated men and women. Living alone increased the odds of sports participation in women, but not in men. Most physical health-related factors were only selectively associated with either SA or DA, in men or women, respectively. The need for a walking aid was the only factor that consistently lowered the odds of being active in both activity domains and sexes.

**Conclusions:**

This exploratory study delivers reliable and relevant data on the participation in and correlates of sporting and domestic activities of community-dwelling older adults for whom there had previously been only limited information at a population level in Germany. Findings are discussed and implications for epidemiological research and health promotion practice are provided.

## Background

The phenomenon of the demographic change is well known [1]. In the future, it will increasingly challenge health care systems in terms of financial and personal resources. Thus, the health status of the older population will be of particular importance. Physical inactivity constitutes a major health risk [2], whereas being sufficiently physically active has the potential to preserve or improve health and functioning [3], and in turn foster older adults' independence and health-related quality of life. Recently, extensive efforts have been made to summarize the compelling evidence regarding the multifaceted health benefits of regular physical activity and exercise, even for previously sedentary and chronically diseased older people [4,5]. In light of the new evidence, the updated "2008 Physical Activity Guidelines for Americans" [6] emphasize that health benefits are not only derived from sports and exercise. Lifestyles that are generally active, including domestic activities such as housework and gardening, are considered to be beneficial as well. In order to derive reasonable health promotion and intervention strategies, both insights into physical activity patterns of specific population subgroups and a deeper understanding of the determinants of physical activity are needed.

To date, little is known about the physical activity behavior of older adults in Germany. The German National Health Interview 2003 [7] and the German annual telephone survey 2009 (GEDA) [8] assessed sporting activity for the adult population. The low levels of sporting activity for older adults revealed by these studies are consistent with international epidemiological research [9-16]. However, studies focusing exclusively on sports and exercise overlook activity domains that are particularly important for people in old age. Research explicitly exploring physical activity patterns in the elderly population identified heavy housework and gardening as among the most common activities performed [13,14,17-21]. It has been suggested that these everyday activities compensate for the decline in sports and exercise observed with increasing age. However, for Germany, so far only one study has been published to date that considers different activity domains that are relevant for older adults [22].

The participation in different physical activities is considered to be influenced by a complex interaction of sociodemographic, physical, psychological, social, environmental and sociopolitical factors [23,24]. Although many studies examined potential determinants of physical activity, little research focused on older adults [24]. Therefore, observations regarding the predictors of physical activity in this population group are rare and those that exist are largely inconsistent, especially when relating to different categories of physical activity.

Physical health status is one of the most important determinants of physical activity behavior in aging. Poor health is the barrier to physical activity cited most often by older adults [25,26] and has been shown to predict the initiation and maintenance of physical activity in this population [24]. However, no detailed analyses regarding the influence of physical health-related factors on physical activity exist. Most studies that have been published either considered the influence of the mere number of chronic conditions on physical activity [19,27], or only took a limited number of (chronic) physical ailments into account [15,28,29]. We hypothesize that specific chronic conditions or specific physical health-related factors such as pain or limited lower body function may differently impact physical activity, i.e. sporting or domestic activity. Knowledge about the associations between these specific factors and physical activity may facilitate targeted interventions in health care.

Since gender is one of the most consistent factors influencing physical activity [30], it would be reasonable to examine potential determinants of physical activity separately for men and women in order to obtain insights on gender-related activity behavior. Differentiated analyses of different activity domains would help to gain deeper understanding of participation in various activities. However, existing studies examining the potential determinants of physical activity among older adults either focused on one type of activity, studied only men or women, or displayed results for total activity or combined the results for both sexes [11,14,15,17,19-21,27,28,31-33]. To the authors' knowledge, no research has been published to date that simultaneously investigated factors associated with sporting activity and domestic activity in older men and women, respectively.

Consequently, the first aim of the study was to analyze engagement in sporting and domestic activities of older men and women in Germany. The second aim was to explore factors - physical health-related factors in particular - associated with performance of sporting and domestic activities separately by sex.

## Methods

### Design and participants

The "German epidemiological trial on ankle brachial index" (getABI) is a prospective, large-scale observational study. Its design and methods have been described elsewhere in greater detail [34,35]. In brief, each of the participating 344 general practitioners consecutively recruited on average 20 eligible patients who sought primary health care during a predefined week in October 2001 and fulfilled the inclusion criteria (age ≥ 65 years, being legally competent and able to co-operate appropriately, and providing written informed consent). The only exclusion criterion was life expectancy ≤ 6 months. A total of 6,880 community-dwelling primary health care patients were included in the study. Within the 7-year follow-up period, 1,302 patients died. The remaining 5,578 patients were contacted by letter and by one telephone call to evaluate their willingness to participate in the computer-assisted telephone interview at the 7-year follow-up. One hundred and ninety-six patients were unable to participate in the interview; another 3,445 patients did not participate in the interview for several other reasons (not reachable, did not want to be contacted by telephone, refused to participate in the telephone interview). In the end, a sample of 1,937 patients (response rate 34.7%) took part in the telephone interviews at the 7-year follow-up. A comparison of these participants to non-participants revealed the following significant differences: participants were younger at baseline (median age (range): 70 (65-85) years vs. 72 (65-91) years), were more often male (46.7% vs. 35.7%), and were better educated (qualification higher than basic secondary school: 40.2% vs. 27.1%). Results reported in this paper predominantly refer to cross-sectional data collected during the 7-year follow-up interviews.

The study was approved by the University of Heidelberg and the University of Bochum (Germany) ethics committees, and was conducted according to the "Good Epidemiological Practice" recommendations issued by the "German Working Group Epidemiology" [36].

### Outcomes

#### Physical activity

As part of the telephone interviews of the 7-year follow-up subjects were surveyed on their everyday physical activity patterns using the PRISCUS Physical Activity Questionnaire (PRISCUS-PAQ) [37]. To ensure appropriateness of physical activity-related questions, participants were initially asked about their walking ability in the week prior to the interview. Evaluation revealed that five interviewees were either wheelchair-bound or bed-ridden, and were thus not queried about their physical activity. To guarantee valid data concerning usual physical activity patterns, those 137 participants who specified that they had been markedly restricted in their physical activities in the preceding week due to a current incident (e.g. fall, accident, severe flu) were also excluded from physical activity questions. One participant who did not provide specific information for either of the two questions was also excluded. In the end, 1,794 patients could be interviewed concerning their everyday physical activity. They did not vary in age and sex from participants who were excluded.

Detailed information on the development, the structure and the reliability of the 10-item PRISCUS-PAQ has already been published [37]. In summary, all questions referred to the prior week except for the item on gardening, which referred to the prior two weeks to account for short-term weather variations. Participants had to state the number of days for each activity and the mean duration for activities performed. Time per week (hh:mm) was then calculated for the single items and for activity categories.

With regard to the purpose of the study, the items bike-riding, exercises and/or strength training, organized sports groups, and other sporting and leisure activities that caused sweating were subsumed under the category "sporting activities." Heavy housework and gardening will be referred to as "domestic activities."

The new 2008 Physical Activity Guidelines recommend an accumulation of at least 150 minutes of moderate-intensity activity in order to bring about health benefits [6]. This benchmark was used for binary categorization of respondents as active (≥ 2.5 hrs/wk) and insufficiently active or inactive (< 2.5 hrs/wk).

### Covariables

The main covariables were sociodemographic and physical health-related factors. Season (interview date) was included as an environmental variable.

#### Sociodemographic factors

At baseline, the general practitioner documented the participants' sex, date of birth and educational level (no qualification - completed basic secondary school - vocational school - university entrance qualification). During telephone interviews, participants were surveyed about their native country (5-year follow-up) as well as the number of persons living in the same household (7-year follow-up).

#### Cardiovascular risk factors

Waist circumference was measured by the general practitioner using a standard protocol at the 5-year and the 7-year follow-up of the getABI cohort. Waist circumference at 7-year follow-up was used for analysis. If values were missing, waist circumference at 5-year follow-up was used. The current smoking status was documented at baseline (smoking no/yes).

The following covariables were assessed in the course of the 7-year follow-up telephone interviews:

#### Chronic conditions

Participants were asked whether they suffered from any of the following chronic diseases (no/yes): arterial hypertension, coronary heart disease, myocardial infarction, chronic heart failure (CHF), diabetes mellitus, peripheral arterial disease, chronic obstructive pulmonary disease (COPD), arthritis (degenerative or rheumatic) or osteoporosis.

#### Walking ability, falls and pain

Walking ability was assessed by asking participants whether they needed a walking aid (no aid - cane - rollator - wheelchair-bound - bed-ridden). Falls were defined as "an unexpected event in which the participants come to rest on the ground, floor, or lower level" [38]. Respondents reported falls within the previous 12 months (no/yes), and pain within the past 3 months (no/yes).

#### Season

To encode the seasons, the interview month was extracted from the interview date. Telephone interviews held in April to September were coded as "spring/summer", those held in the remaining months were coded as "autumn/winter".

### Statistical analysis

In order to describe the getABI patients who were analyzed, the univariate distribution of categorical variables is presented in terms of absolute frequencies, percentages and p-values from Pearson's chi-square test (see Table 1). Analyses of physical activity patterns are descriptive. They include percentages for engagement in domestic or sporting activities on at least one day in the week prior to the interview, median time per week spent on the respective activities, and percentages for those performing these activities for at least 2.5 hrs/wk.

**Table 1:** Participants' characteristics at time of the 7-year follow-up by sex

	Men (n = 780)		Women (n = 830)		p-value
		
	n	%	n	%	
**Outcomes**					
Sporting activities ≥ 2.5 hours per week	325	41.7	256	30.8	< .001
Domestic activities ≥ 2.5 hours per week	426	54.6	566	68.2	< .001

**Sociodemographic factors**					
Age ≥ 80 years	246	31.5	293	35.3	.110
Place of birth Germany	704	90.3	759	91.4	.408
Educational level higher than basic secondary school	348	44.6	323	38.9	.020
Living alone	126	16.2	449	54.1	< .001

**Cardiovascular risk factors**					
Currently smoking (baseline)	57	7.3	54	6.5	.526
Waist circumference ^a^: women ≥ 88 cm; men ≥ 102 cm	439	56.3	582	70.1	< .001

**Chronic conditions**					
Hypertension	480	61.5	559	67.3	.015
CHD and/or myocardial infarction	265	34.0	148	17.8	< .001
Chronic heart failure	159	20.4	146	17.6	.153
Diabetes mellitus	213	27.3	188	22.7	.031
Peripheral arterial disease	101	12.9	80	9.6	.036
Chronic obstructive pulmonary disease	79	10.1	96	11.6	.354
Arthritis (degenerative or rheumatoid)	239	30.6	318	38.3	.001
Osteoporosis	37	4.7	171	20.6	< .001

**Other health-related factors**					
Need for walking aid	110	14.1	147	17.7	.048
Falls (past 12 months)	131	16.8	208	25.1	< .001
Pain (past 3 months)	357	45.8	495	59.6	< .001

**Environmental factors**					
Season: spring/summer	486	62.3	536	64.6	.334

CHD = Coronary heart disease

^a ^Waist circumference at 7-year follow-up was used for analysis. If this value was missing, waist circumference at 5-year follow-up was used.

Multivariate logistic regression analyses were done to assess the odds of engagement in sporting and domestic activities for at least 2.5 hrs/wk associated with sociodemographic variables, cardiovascular risk factors, chronic conditions, walking ability, falls, pain, and season. Covariables (for binary categorization see Table 2) were simultaneously entered in the four models (for each activity domain and sex, respectively). Participants with missing values in the outcomes or covariables were excluded from analyses. Evaluation of differences between cases with complete and incomplete data was performed by means of the chi-square test (Fisher's exact test). Within the logistic regression analyses, the 95% confidence interval (95% CI) was calculated for each odds ratio (OR).

**Table 2:** Multivariate analyses of factors associated with sporting activities and domestic activities in men and women (active [≥ 2.5 hours/week] vs. inactive)

	Sporting activities				Domestic activities			
	
	Men		Women		Men		Women	
	
	% active^†^	Adjusted OR^# ^[95% CI]	% active^†^	Adjusted OR^# ^[95% CI]	% active^†^	Adjusted OR^# ^[95% CI]	% active^†^	Adjusted OR^# ^[95% CI]
**Sociodemographic factors**								
Age								
< 80 years	46.8	1	34.6	1	57.3	1	73.7	1
≥ 80 years	30.5	0.52 [0.36;0.73]*	23.9	0.55 [0.39;0.78]*	48.8	0.83 [0.59;1.17]	58.0	0.62 [0.44;0.88]*
Place of birth								
Outside Germany	51.3	1	31.0	1	50.0	1	59.2	1
Germany	40.6	0.61 [0.37;1.01]	30.8	1.02 [0.59;1.78]	55.1	1.19 [0.72;1.96]	69.0	1.47 [0.85;2.53]
Education level (qualification)								
No qualification or basic secondary school	42.6	1	28.6	1	60.0	1	72.6	1
Higher than basic secondary school	40.5	0.87 [0.64;1.19]	34.4	1.30 [0.95;1.80]	48.0	0.58 [0.43;0.79]*	61.3	0.68 [0.49;0.94]*
Living situation								
With at least one other person	42.7	1	27.0	1	54.9	1	73.2	1
Living alone	36.5	0.82 [0.54;1.24]	34.1	1.68 [1.22;2.31]*	53.2	0.97 [0.64;1.46]	63.9	0.75 [0.53;1.04]

**Cardiovascular risk factors**								
Currently smoking (baseline)								
No	42.7	1	31.8	1	53.9	1	69.6	1
Yes	28.1	0.45 [0.24;0.84]*	16.7	0.42 [0.19;0.89]*	63.2	1.25 [0.69;2.23]	48.1	0.38 [0.21;0.69]*
Waist circumference^a^								
Women < 88 cm; men < 102 cm	44.3	1	33.1	1	56.3	1	65.3	1
Women ≥ 88 cm; men ≥ 102 cm	39.6	0.82 [0.61;1.12]	29.9	0.97 [0.69;1.37]	53.3	0.88 [0.65;1.19]	69.4	1.30 [0.91;1.84]

**Chronic conditions**								
Hypertension								
No	43.7	1	32.1	1	53.7	1	69.0	1
Yes	40.4	0.88 [0.64;1.20]	30.2	1.03 [0.73;1.43]	55.2	1.08 [0.79;1.48]	67.8	1.02 [0.72;1.44]
CHD and/or myocardial infarction								
No	41.6	1	30.4	1	53.0	1	67.9	1
Yes	41.9	1.19 [0.86;1.65]	33.1	1.35 [0.89;2.05]	57.7	1.35 [0.98;1.87]	69.6	1.31 [0.85;2.04]
Chronic heart failure								
No	43.6	1	31.0	1	55.6	1	70.8	1
Yes	34.0	0.74 [0.50;1.10]	30.1	1.03 [0.68;1.55]	50.9	0.93 [0.64;1.37]	56.2	0.60 [0.40;0.89]*
Diabetes mellitus								
No	44.3	1	33.3	1	55.9	1	69.8	1
Yes	34.7	0.67 [0.47;0.96]*	22.3	0.63 [0.42;0.94]*	51.2	0.81 [0.57;1.14]	62.8	0.82 [0.60;1.22]
Peripheral arterial disease								
No	42.7	1	31.9	1	54.8	1	69.1	1
Yes	34.7	0.89 [0.55;1.43]	21.3	0.65 [0.36;1.20]	53.5	1.00 [0.63;1.61]	60.0	0.89 [0.51;1.53]
Chronic obstructive pulmonary disease								
No	43.2	1	31.2	1	55.3	1	68.7	1
Yes	27.8	0.55 [0.32;0.94]*	28.1	0.80 [0.48;1.31]	48.1	0.68 [0.41;1.12]	64.6	0.91 [0.55;1.49]
Arthritis (degenerative or rheumatoid)								
No	42.0	1	28.9	1	56.2	1	68.4	1
Yes	41.0	1.08 [0.76;1.52]	34.0	1.35 [0.97;1.88]	51.0	0.94 [0.66;1.32]	67.9	1.20 [0.85;1.70]
Osteoporosis								
No	41.9	1	29.4	1	54.4	1	69.3	1
Yes	37.8	0.90 [0.44;1.86]	36.3	1.48 [1.01;2.17]*	59.5	1.29 [0.62;2.69]	63.7	0.87 [0.59;1.30]

**Other health-related factors**								
Need for walking aid								
No	44.8	1	33.7	1	57.6	1	74.1	1
Yes	22.7	0.46 [0.28;0.78]*	17.7	0.46 [0.28;0.75]*	36.4	0.42 [0.26;0.68]*	40.8	0.28 [0.18;0.42]*
Falls (past 12 months)								
No	43.3	1	30.9	1	55.3	1	69.3	1
Yes	33.6	0.81 [0.53;1.24]	30.8	1.07 [0.74;1.54]	51.1	1.02 [0.68;1.54]	64.9	1.06 [0.73;1.53]
Pain (past 3 months)								
No	40.7	1	34.6	1	56.0	1	71.6	1
Yes	42.9	1.22 [0.89;1.67]	28.3	0.68 [0.49;0.95]*	52.9	1.05 [0.76;1.44]	65.9	0.78 [0.55;1.11]

**Environmental factors**								
Season								
Autumn/winter	41.5	1	28.6	1	40.5	1	60.5	1
Spring/summer	41.8	1.04 [0.76;1.43]	32.1	1.14 [0.82;1.59]	63.2	2.73 [2.00;3.73]*	72.4	1.67 [1.20;2.32]*

CHD = Coronary heart disease; OR = Odds ratio; CI = Confidence interval

^† ^for at least 2.5 hours/week

^# ^Adjusted for the other variables in the table as well as for activity in the other domain

^a ^Waist circumference at 7-year follow-up was used for analysis. If this value was missing, waist circumference at 5-year follow-up was used.

* 95% Confidence interval not including 1.

## Results

### Participants

Of the 1,794 participants, 184 were excluded due to incomplete data. A slightly higher number of men delivered complete data for analyses (92.5% vs. 87.3%). Men who answered all questions more often had a higher level of education than those who did not answer completely (44.6% vs. 22.0%). Women with complete data more often suffered from at least one chronic condition compared to those who failed to answer all questions (89.6% vs. 82.5%). Cases with complete and incomplete data did not substantially differ with regard to age, cardiovascular risk factors, walking ability, falls, pain or season.

A total of 1,610 primary health care patients (51.6% women) with a median age of 77 (range 72-93) years were ultimately included in the analyses. These participants' characteristics are displayed by sex in Table 1.

### Physical activity patterns

**Figure 1:**
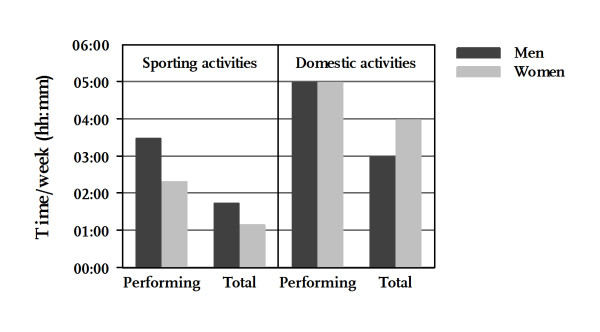
**Median time per week (hh:mm) spent in sporting activities and domestic activities by sex**. Median values in activity categories are depicted (1) for participants performing the respective activities at least once a week prior to the interview (Performing sporting activities: men 67.3%, women 66.3%; performing domestic activities: men 79.1%, women 86.6%) as well as (2) for all respondents including those not performing the respective activities and being assigned a null for time/week (Total; men: n = 780, women: n = 830).

Physical activity patterns of men and women with regard to performance rates and median time per week (hh:mm) are shown in Figure 1. While the rates of engagement in sporting activities on at least one day in the week prior to the interview were comparable between men and women (67.3% vs. 66.3%), men spent more time per week in these activities than women did (03:30 vs. 02:20). In contrast, median time per week for those performing domestic activities was equal between the sexes although fewer men than women stated that they had done heavy housework and/or gardening in the previous week (79.1% vs. 86.6%).

To appraise the amount of physical activity of the total samples of men and women, median time per week was also been evaluated for all respondents including those who did not perform the respective activity, and thus were assigned a null for time per week. Altogether, men engaged more in sporting activities than women (01:45 vs. 01:10), whereas women performed more domestic activities per week than their male counterparts (04:00 vs. 03:00).

Accordingly, while more male than female participants reached a minimum of at least 2.5 hours/week (hrs/wk) of sporting activities (41.7% vs. 30.8%), more women than men reached this benchmark with regard to domestic activities (68.2% vs. 54.6%).

### Correlates of sporting and domestic activities

Factors independently associated with sporting and domestic activities in men and women are shown in Table 2. Higher age and being a smoker was associated with decreased physical activity among men and women, except for men performing domestic activities. Males and females with a higher level of education had a lower domestic activity level, while there was no association with sporting activity. Living alone increased the chance of females engaging in sporting activities by nearly 70%.

Regarding health-related factors, walking ability was the only factor consistently associated with reduced odds of being active in both activity domains and both sexes. Diabetes mellitus was found to lower the odds of performing sporting activities in men and women. Chronic heart failure was negatively associated with domestic activity in women, while chronic obstructive pulmonary disease was negatively associated with sporting activity in men. While female participants with pain less frequently reached the minimum amount of 2.5 hrs/wk of sporting activity, those with osteoporosis had higher odds of sports participation, though associations were weak.

Finally, with respect to the environmental factor considered in the analyses, season was most strongly associated with activity in the house and/or garden, while it was unrelated to sporting activity. In both sexes, the following factors did not prove to be independently associated with either activity domain: place of birth, waist circumference, hypertension, peripheral arterial disease, arthritis, history of falls.

## Discussion

This study evaluated physical activity patterns of older men and women in Germany, and explored correlates of sporting and domestic activity.

### Physical activity patterns

Analyses revealed gender-related differences regarding time spent in sporting activities or domestic activities. Generally, this finding is in line with national and international data on physical activity patterns in elderly [7,8,11,16,22,28].

Luehrmann et al. [22] investigated energy expenditure within a longitudinal study in an elderly population in Germany (GISELA). They assessed physical activity patterns via questionnaire and presented mean hours per day (hrs/d) spent in different activities. The study revealed that women engaged more in housework and gardening than men (women 03:40 hrs/d, men 02:06 hrs/d), whereas male participants spent more time in sporting activities (men 00:46 hrs/d, women 00:30 hrs/d), supporting findings in the getABI cohort. However, when our result are considered from time/week to time/day, much lower levels of physical activity are found in the getABI cohort in both activity domains and sexes (see Figure 1). Several aspects may have led to these discrepancies: 1. The GISELA study presented time per day as mean values, while we decided to present the median time due to skewed data distribution in the direction of low values; mean values obviously would have been higher. 2. Regarding the category of domestic activities, Luehrmann et al. did not differentiate between light and heavy housework. In the present study, both light and heavy housework were assessed, but only heavy housework was considered for analyses accounting for merely one-quarter of total time spent on housework. 3. The GISELA study assessed time for housework and gardening per day. In contrast, the PRISCUS-PAQ first asked about the number of days on which the activity was performed in the previous week and then for mean time spent in the respective activities. Calculating time per week will consequently yield lower duration for all participants who specified that they had performed the activity on fewer than 7 days. Comparing our results to others in this field of research reveals several methodological issues that merit consideration. Specifically, the use of differing questionnaires can be problematic.

In addition, ever changing physical activity guidelines can at times affect comparability across studies when they are used as a method of categorization. Previously researchers aligned their categorization with the American College of Sports Medicine and American Heart Association (ACSM/AHA) recommendations for adults [39] or older adults [3], recommending at least 30 minutes of moderate-intensity activity on five, preferably on all days of the week. Since 2008, the updated guidelines released by the U.S. Department of Health and Human Services have allowed an accumulation of 150 minutes of moderate-intensity physical activity. The dilemma is that applying differing sets of recommendations to categorize subjects as active or inactive yields different results [12,40,41]. Researchers from the U.S. Centers for Disease Control and Prevention [40] analyzed data of the 2007 Behavioral Risk Factor Surveillance System (BRFSS), applying both above-mentioned sets of recommendations. 64.5% of respondents met the 2008 Guidelines, while only 48.8% of the same respondents were classified as sufficiently active according to the older benchmark of getting physical activity at least 5 times a week for 30 minutes or more [40]. This methodological problem also becomes evident in the following comparisons of our results to the results from existing German studies that assessed prevalence of physical activity in old age.

Using the German National Health Interview 2003 [7], researchers revealed the following rates of sports engagement for the age group 70-79 years: men 29.9%, women 22.2%; at least 2 hrs/wk: men 15.5%, women 17.5%. GEDA 2009 [8] showed that performance rates have increased. In the age group 65 years and older, 35.2% of men and 29.2% of women engaged in sports for a minimum of 2 hrs/wk. Although participants in the present study needed to accumulate at least 2.5 hrs/wk of sporting activities, rates in the getABI cohort were higher (men 41.7%, women 30.8%). These differing performance rates may be attributed to the way questions are posed. In the getABI study, respondents were asked for activities such as bike-riding, exercises or strength training, participation in organized groups, or performance of other leisure activities that caused sweating, the German National Health Interview and GEDA 2009 assessed sporting activity based on a single item: "How often did you perform sports in the past week?" and "Think about the past three months. Did you perform sports during this period?" It may be assumed that people in old age interpret the term "sports" quite differently. Various activities, such as gymnastics or dancing may not always be considered to be sporting activities. As a consequence, this may have led to an underreporting of performing sporting activities in the two German studies cited. Finally, despite methodological problems hampering comparisons between studies, the findings from the current study fit into an overall epidemiological picture of physical activity in old age.

### Correlates of sporting and domestic activity

#### Sociodemographic factors

According to the updated review on correlates of adults' participation in physical activity [30], higher age is consistently associated with lower physical activity. This was also the case in our study, although the association between higher age and lower domestic activity in men was not significant. In the present study, age was not associated with domestic physical activity in men. However, even when age is found to be independently associated with lower activity, as is the case here for women, age itself is never the factor influencing physical activity. There are rather underlying physiological (e.g. decreased physical functioning), psychosocial (e.g. attitude towards physical activity in old age, fear of crime or injury, social support) or environmental factors (e.g. no place to sit down, no/uneven sidewalks) explaining decreasing activity with increasing age.

Education has been shown to be strongly positively associated with physical activity [30]. However, the present study found contradicting results. While there was no significant association between educational level and sporting activity in our study, domestic activities were significantly less frequently performed by more highly educated men and women. A study in older women from the U.K. [14] confirmed that belonging to the managerial and professional class was associated with doing significantly less heavy housework compared to women from the intermediate and working class. We speculate that better education may result in higher income, and thus the possibility to pay others to do household chores, or the gardening is plausible.

Living alone was a factor independently associated with increased sporting activity in women. Comparisons with other findings are difficult for several reasons. Researchers usually assessed marital status [11,14,31,32,42], which precludes statements on whether the older person is living on his/her own, or with others. Furthermore, there is no German study available regarding the association between living alone and the level of sporting activity in elderly men and women. One study among Swedish elderly assessed the level of outdoor recreational physical activity - comprising sporting activities, gardening, and walking - and associated factors [43]. In contrast to our results, there was no independent association of living alone and outdoor recreational physical activity level in either men or women. Another study investigating overall physical activity in 72 older Australian women found those living alone to be significantly more active [44]. Although it did not focus on sporting activities and did not study both sexes, this study supports the findings in female getABI patients. The patient characteristics in Table 1 show that many more female getABI patients than male getABI patients stated that they lived alone (54.1% vs. 16.2%). Consistent with our analyses is the fact that 49% of women but only 17% of men aged 65 years and older were living alone in Germany in the year 2004 [45]. The Contribution to Federal Health Reporting entitled Health and Morbidity in Old Age [46] pointed out that the living situation for older women turns out to be fundamentally different compared to older men. With increasing age, the rate of widowed women rises dramatically, since at the age of 80 years and older, three-quarters of all women are widowed. The rate of older adults who are single and divorced is also higher in women than in men [46]. In sum, more older women than men have to cope with the situation of living alone. However, compared to men living alone, older women appear to more actively participate in social life, to be more motivated and engaged in arranging their future phase of life, and more often consider reorientation [47]. It is thus possible that engagement in sporting activities, which may go along with building (new) social networks, is one way for women to cope with the (often long-lasting) situation of living alone.

#### Season

The factor most strongly associated with increased odds of being active in the house and/or garden was being interviewed in spring or summer (SS) as compared to autumn or winter (AW). This was true for both men and women. As expected, these associations in the category of domestic activities are mainly attributable to the seasonality of gardening. While the rate of those doing heavy housework on at least one day in the week prior to the interview did not substantially differ between the two survey periods (SS: 66.8%; AW: 69.4%), there was a remarkable difference in rates of gardening between seasons (SS: 66.3%; AW: 32.7%). Unexpectedly, there was no association between season and sporting activities in the getABI cohort. In the literature, there is increasing evidence that physical activity behavior in general is subject to seasonal variations. Twenty-seven out of 37 epidemiological studies reviewed by Tucker and Gilliland [48] revealed an impact of season, and subsequently weather, on physical activity behavior of (older) adults. The highest physical activity levels were registered in the spring and summer months. The study by Sumukadas et al. [49] examined the impact of weather on objectively measured physical activity in functionally impaired elderly on a day-to-day basis. The older persons' physical activity peaked in the summer months, supporting the results of the review. Day length, maximum temperature and sunshine duration were identified as independently associated with daily activity, both at the intra- and inter-individual level. Especially for older adults, extreme cold, slippery ground due to precipitation, or early dusk (impaired vision, fear of crime) may constitute barriers to outdoor physical activity in the winter months. Finally, Shephard and Aoyagi [50] pointed out that as physical activity fluctuates in the course of the year, so do parameters of physical fitness and health.

#### Chronic conditions and other physical health-related factors

We hypothesized that specific chronic conditions and other physical health-related factors may have a different impact on sporting and domestic activity level. Analyses revealed that only the need for a walking aid was consistently associated with lower physical activity in both activity categories and sexes. These findings are supported by an Australian study investigating recreational physical activity in 1,500 healthy older women. The proportions of women using a cane were significantly different by activity level, with the highest proportion in the sedentary group (14.2%) and the lowest proportion in the high activity group (3.3%) [51]. Gait unsteadiness due to a loss of lower-body strength and balance, and resulting fear may be an explanation for the decreased activity in older people who depend on a walking aid. Several studies showed that using walking devices is independently associated with being more fearful of falling [52-54]. Fear of falling has in turn been reported as a barrier to physical activity [26,51,55,56]. In those with moderate or severe mobility restriction, fear of falling has been even more frequently reported as a barrier to exercise [57]. It has been shown that fear-induced activity reduction [58] or the need for a walking aid [59], and the tendency to adopt a more sedentary lifestyle accelerate functional deconditioning and disability.

The chronic conditions and the remaining physical health-related factors, and their associations with physical activity levels revealed a quite inconsistent picture. Single conditions were found to be associated with one activity category in either women or men, respectively. Only diabetes mellitus was associated with reduced sporting activity in both sexes. Hays et al. [60] studied correlates of overall physical activity in a sample of older diabetics and showed that motivational barriers, i.e. lack of will power, interest and time, were strongly associated with reduced physical activity among these people. It may thus be considered particularly challenging to increase physical activity and especially sporting activity among diabetics.

The cross-sectional design of this study precludes decisions on the direction of associations. However, we speculate that compared to a condition such as hypertension that does not become noticeable during physical activity, the perceptible effects of conditions such as CHF and COPD (e.g. extreme shortness of breath, accelerated fatigue) may cause a reduction in physical activity, like that found for domestic activities in women or sporting activities in men in our study. Additionally, pain may obviously limit the willingness to engage in sporting activities, as was the case for women. A difference in pain intensity as well as disease severity between men and women may explain the inconsistent findings with regard to these health-related conditions and sex. Surprisingly, having osteoporosis increased sporting activity in women. The reasons for this finding are unclear. Further investigations regarding the relevance of health-related factors for engagement in different physical activities are necessary to better understand potential underlying mechanisms of activity restriction based on specific diseases.

### Strengths and weaknesses

The assessment of physical activity in epidemiological research has known limitations [61]. Self-report is subject to bias (recall of activities performed in the past, social desirability bias). However, so far it has been the only way to obtain information on physical activity in different activity domains at low cost and in large populations [62].

Cross-sectional studies have been suggested to be an efficient and empirical means of screening a broad range of potential correlates of physical activity [63]. Associations in fact do not allow causal inferences, but provide a basis for generating hypotheses. The strength of the present study is the inclusion of a broad range of potential, particularly health-related determinants to explore the associations with two categories of activities separately by sex. The omission of measures of perceived health status limits the informative value of our results. We did not consider a broader range of psychological, socio-cultural or environmental variables that have been shown to influence physical activity. This is a limitation of our study.

The response rate of the 7-year follow-up telephone interview was 34.7%. As expected, participants were younger and better educated compared to non-participants. Furthermore, it can be assumed that the willingness and ability to continue to participate in a longitudinal trial after 7 years is higher in healthier persons. Participants who had moved to a nursing home during the follow-up period were no longer able to participate. Therefore, there is most likely a selection towards the fitter patients from baseline to the 7-year follow-up in the getABI cohort. Our results should thus be regarded as relevant for a population of relatively fit seniors who are still able to visit their GP and take part in a telephone interview.

## Conclusion

Despite the above-mentioned limitations, this study delivers reliable and relevant data on the engagement in, and correlates of sporting, and domestic activities of community-dwelling older adults for whom there had been little information at a population level in Germany so far.

### Implications for epidemiological research

Research on physical activity behavior of older men and women should include the investigation of engagement in diverse physical activities. When merely asking about total physical activity, important information is lost. In our study for instance we might have obtained comparable total physical activity levels for men and women, overlooking women's decreased participation in sporting activities, which has important implications for health promotion practice (see below).

Despite the input of the present study concerning correlates of physical activity, knowledge about the influence of education, living situation, smoking, diverse health-related factors, and season on different categories of physical activity remains inconsistent and unclear. Differentiated epidemiological analyses should attempt to clarify how these and also other factors impact the engagement of older adults in diverse activities such as sports, housework, or gardening, since this could have implications for health promotion practice. When studying the potential impact of physical health-related conditions on physical activity, severity and subjective impairment resulting from each condition should be included. Moreover, the variability of activity prevalence rates in the course of the year has to be considered in the surveillance of physical activity. Finally, a national consensus on a standardized instrument to assess physical activity in older adults is needed to increase comparability of research.

### Implications for health promotion practice

The finding that older men engaged in sporting activities more often while women performed more domestic activities corresponds to conventional role assignment. It has been suggested that domestic activities may compensate for low participation in sporting activity. However, we suppose that performance of heavy housework may not yield the same benefits for health and psycho-physical well-being as participation in leisure sporting activities. Health care providers are thus challenged to increase older women's interest of and motivation for sports and exercise, despite a potential lack of experience with practice and higher level of involvement in 'family responsibilities'.

The seasonality of physical activity behavior is of particular relevance for health promotion practice. Health care professionals should sensitize older adults to the seasonal variations in physical activity behavior and the implications for health and functioning. Alternatives to outdoor physical activities should be discussed for the winter months to ideally maintain the physical activity level throughout the year.

Finally, the present study showed that older people who suffer from specific chronic diseases such as CHF and COPD, pain, or impaired mobility probably reduce their sporting or domestic physical activities due to the perceptible impacts of these conditions. They are thus in danger of accelerated functional decline and social withdrawal. Although it may be considered challenging, health care providers should particularly approach older adults with noticeable health and functional impairments in order to motivate them to engage in regular physical activity. These people would greatly profit from individually adapted physical activity programmes in terms of preventing progression of their disease and disability, and preserving their independence and health-related quality of life.

## Conflict of interest

The authors declare that they have no competing interests.

## Authors' contributions

TH and PP obtained the research grant for the project "Physical activity, multimorbidity and polypharmacy in the elderly" within the PRISCUS research cooperation [64] and initiated the specific data collection in the getABI cohort. TH coordinated the project. AM and TH conceived the research question and the statistical design of the present study. AM edited the data and performed the statistical analyses. RKM participated in data preparation and double-checked the statistical analyses. AM, TH and UT interpreted the data. AM drafted the manuscript. All authors revised the manuscript critically for important intellectual content. All authors approved the version to be published.

## Pre-publication history

The pre-publication history for this paper can be accessed here:

http://www.biomedcentral.com/1471-2458/11/559/prepub
